# LB14. Efficacy and Immunogenicity of an Ad26.RSV.preF-based Vaccine in the Prevention of RT-PCR-confirmed RSV-mediated Lower Respiratory Tract Disease in Adults Aged ≥65 Years: A Randomized, Placebo-controlled, Phase 2b Study

**DOI:** 10.1093/ofid/ofab466.1650

**Published:** 2021-12-04

**Authors:** Ann R Falsey, Kristi Williams, Efi Gymnopoulou, Stephan A Bart, John E Ervin, Arangassery Rosemary Bastian, Joris Menten, Els De Paepe, Hilde de Boer, Sjoukje Vandenberghe, Eric Chan, Jerald Sadoff, Macaya Douoguih, Benoit Callendret, Christy Comeaux, Esther Heijnen

**Affiliations:** 1 University of Rochester, Rochester, New York; 2 Janssen Research and Development, Spring House, PA, USA, Spring House, Pennsylvania; 3 Janssen Infectious Diseases, Beerse, Antwerpen, Belgium, B-2340, Beerse, Antwerpen, Belgium; 4 Optimal Research, LLC/Synexus Clinical Research/AES, Woodstock, MD, USA, Woodstock, Maryland; 5 Alliance for Multispecialty Research - KCM, KANSAS CITY, Missouri; 6 Janssen Infectious Diseases and Vaccines, Leiden, Zuid-Holland, The Netherlands, 2333, Leiden, Zuid-Holland, Netherlands; 7 Janssen Infectious Diseases, Beerse, Belgium, Beerse, Antwerpen, Belgium; 8 Janssen-Cilag, Tilburg, The Netherlands, Tilburg, Noord-Brabant, Netherlands; 9 Janssen Global Services, LLC, Raritan, NJ, USA, Raritan, New Jersey; 10 Janssen Vaccines and Prevention, Leiden, Netherlands, Leiden, Zuid-Holland, Netherlands

## Abstract

**Background:**

Respiratory syncytial virus (RSV) can cause serious lower respiratory tract disease (LRTD) in older adults. Despite a high burden of disease, there is currently no licensed vaccine for RSV. Here, we report the primary efficacy and immunogenicity results from a Phase 2b proof-of-concept trial of an Ad26.RSV.preF-based vaccine for the prevention of RSV-mediated LRTD in adults aged ≥65 years.

**Methods:**

CYPRESS (NCT03982199) is a randomized, double-blind, placebo-controlled Phase 2b trial. Adults ≥65 years of age were randomized 1:1 prior to the RSV season to receive an Ad26.RSV.preF-based vaccine or placebo. Symptoms of acute respiratory infection (ARI) were collected through an RSV-specific patient-reported Respiratory Infection Intensity and Impact Questionnaire (RiiQ) and/or by a clinician assessment until the end of the RSV season. The primary endpoint was the first occurrence of RT-PCR-confirmed RSV-mediated LRTD according to any of 3 case definitions: (1) ≥3 symptoms of lower respiratory tract infection (LRTI), (2) ≥2 symptoms of LRTI, or (3) ≥2 symptoms of LRTI or ≥1 symptom of LRTI with ≥1 systemic symptom. The secondary endpoint was the first occurrence of any RT-PCR-confirmed RSV-mediated ARI. Immunogenicity assessments were performed in a subset of approximately 200 participants.

**Results:**

A total of 5782 participants (2891 in each study arm) received study treatment (92.5% white, 57.7% female, median age 71 years). Vaccine efficacy was 80% (94.2% CI, 52.2–92.9%), 75% (50.1–88.5%), and 69.8% (43.7–84.7%) for case definition 1, 2, and 3, respectively (all *P* values < 0.001). Efficacy for any RSV-mediated ARI was 69.8% (95% CI, 42.7–85.1%). In the vaccine arm of the immunogenicity subset, geometric mean fold increase in antibody titers 14 days after vaccination was 13.5 for RSV neutralizing antibodies and 8.6 for RSV prefusion F-specific binding antibodies. Median frequency of RSV-F-specific INFγ T-cells increased from 34 to 444 SFC/10^6^ PBMC 14 days after vaccination in the vaccine arm; no relevant changes were observed in the placebo arm.

**Conclusion:**

In CYPRESS, the Ad26.RSV.preF-based vaccine was highly effective against RSV-mediated LRTD through the first RSV season and elicited robust humoral and cellular immune responses in adults aged ≥65 years.

**Disclosures:**

**Ann R. Falsey, MD**, AstraZeneca (Individual(s) Involved: Self): Grant/Research Support; BioFire Diagnostics (Individual(s) Involved: Self): Grant/Research Support; Janssen (Individual(s) Involved: Self): Grant/Research Support; Merck, Sharpe and Dohme (Individual(s) Involved: Self): Grant/Research Support; Novavax (Individual(s) Involved: Self): Other Financial or Material Support, Paid DSMB member; Pfizer (Individual(s) Involved: Self): Grant/Research Support **Kristi Williams, PhD**, **Janssen R&D US** (Employee) **Efi Gymnopoulou, MSc**, **Janssen Infectious Diseases BV** (Employee) **Arangassery Rosemary Bastian, PhD**, **Janssen Vaccines & Prevention BV** (Employee) **Joris Menten, n/a**, **Janssen Infectious Diseases BV** (Employee) **Els De Paepe, MSc**, **Janssen Infectious Diseases BV** (Employee) **Hilde de Boer, MSc**, **Janssen-Cilag** (Employee) **Sjoukje Vandenberghe, n/a**, **Janssen Infectious Diseases BV** (Employee) **Eric Chan, PhD**, **Janssen Global Services, LLC** (Employee) **Jerald Sadoff, MD**, **Johnson & Johnson** (Employee, Shareholder) **Macaya Douoguih, MD, MPH**, **Janssen** (Employee) **Benoit Callendret, PhD**, **Janssen Vaccines & Prevention BV** (Employee) **Christy Comeaux, MD**, **Janssen Vaccines & Prevention BV** (Employee) **Esther Heijnen, MD**, **Janssen Vaccines & Prevention BV** (Employee)

